# Screening of vascular pathologies – evidence-based monitoring of prevention efficiency

**DOI:** 10.1186/1878-5085-5-S1-A91

**Published:** 2014-02-11

**Authors:** Ulyana B Lushchyk, Viktor V Novytskyy, Igor P Babii, Lyudmyla S Riabets, Oleg P Kolomiychuk, Ivanna I Legka

**Affiliations:** 1 Research Center Veritas, 31 Obolonska Str., of. 9, Kyiv, 04071, Ukraine; 2 Clinic Victoria Veritas, 4 Williams Str., Kyiv, 03191, Ukraine; 3 Center for Innovative Medical Technologies Veritas IT Med, 4 Williams Str., Kyiv, 03191, Ukraine; 4 Institute for Mathematics of NAS of Ukraine, 3 Tereshchenkivska Str., 01601, Kyiv, Ukraine

## Scientific objectives

Some important aspects in evidence-based medicine have not been included in basic and applied research. The current level of examination of the cardiovascular system requires new analytical approaches for the prevention of cardiovascular diseases, insults and infarctions in people of different age, and diminishing of disability and mortality of vascular critical states.

## Technological approaches

The technology of vascular screening enables not only to make accurate diagnoses but also to treat efficiently that reduces financial burden of a patient and his relatives. The medical technology will be useful in prophylactic and preventive medicine of the world for the health improvement of CVD patients.

## Results interpretation

Regular vascular screening enables to perform diagnostics of microcirculatory disorders, to control efficiency of the therapy almost for every patient non-invasively (psychoneurological, therapeutic, angiological, endocrinological, and cancer neovascularization profiles for the assessment of congenital and acquired vascular anomalies) and during prophylactic exanimations of healthy people who are under influence of external factors, resuscitation departments, insurance medicine and sports medicine.

## Outlook and expert recommendations

The approaches to the analytical estimation of the vascular pipeline on macro- and microlevels allow newly considered problems in the blood supply for organs and systems in the human organism. It enables to estimate the possibility of sanogenic reconstructions in the vascular bed on local, regional, and systemic levels, and the adequacy of such alterations according to the necessities of the ill organism.
